# Purkinje cell activity changes in cerebellar subregions during fear conditioning

**DOI:** 10.3389/fnbeh.2025.1649361

**Published:** 2025-11-12

**Authors:** Johanna Pakusch, Tejas Nair, Thomas Grosch, Melanie D. Mark

**Affiliations:** Behavioral Neuroscience, Ruhr-University Bochum, Bochum, Germany

**Keywords:** fear conditioning, cerebellum, neuronal activity, *c-fos*, TRAP, Purkinje cell

## Abstract

The cerebellum has been recently implicated in modulating fear behavior. The collective activity of Purkinje cells (PCs) across cerebellar regions during fear conditioning remains unexplored. In this brief report we implemented the targeted recombination in active populations (TRAP) system to investigate intracerebellar PC activity during cue dependent fear conditioning in mice. Purkinje cell activity was reduced in areas previously implicated in controlling aversive emotions, which included cerebellar subregions Crus I and II and lobule VI with more pronounced changes in the left cerebellar hemisphere and increases in the lobule VIII subareas in the right hemisphere. Additional PC activity increases related to motor function were observed from the right hemispheric subregions of the paraflocculus and decreases in the left copula pyramidis. These findings underscore the importance of examining the contribution of cerebellar subregions in the context of fear learning, highlighting the potential lateralization of cerebellar fear emotions.

## Introduction

The cerebellum, which is known for its role in motor control, has gained interest for its involvement in emotional learning (Doubliez et al., 2023). Fear conditioning is a form of associative learning in which a neutral stimulus is associated with an aversive event. Subsequently, when exposed to the formerly neutral stimulus alone, it elicits a fear response. Fear learning is part of a normal adaptation to external influences. The limbic system and the medial prefrontal cortex play a role in fear conditioning (Tovote et al., 2015). It is now widely recognized that behaviors are shaped by complex interactions within neuronal networks rather than by single brain regions (Vetere et al., 2017). These networks span multiple brain areas, and recent studies have explored the role of the cerebellum within the fear network (Frontera et al., 2023).

Although the initial indicators of cerebellar involvement in fear learning emerged from early studies on lesions or toxin inactivation, research has transitioned to more precise investigations to examine the function of individual neurons and identify synaptic changes at parallel fiber-PC (Lee et al., 2023) and molecular layer interneuron-PC (Carzoli et al., 2023) synapses after fear conditioning. Past studies have also implicated the influence of climbing fibers (CF) on fear behavior (Xue et al., 2024). Projection-specific interventions of extracerebellar connections during fear acquisition have demonstrated the ability of the cerebellum to shape fear learning and memory (Frontera et al., 2023). However, despite these advances, the basic neuronal activity of cerebellar neurons, specifically PCs in the context of fear acquisition, has not been systematically examined. This leaves a significant knowledge gap to fully comprehend the role of individual cerebellar populations in fear conditioning and provides the opportunity to find intracerebellar regions that are specifically active during fear learning. In this study, we aim to better understand the role of these populations by investigating intracerebellar activity during fear conditioning to unravel its PC activity during emotional learning.

## Materials and methods

### Mice

The local ethics committee (Bezirksamt Arnsberg) and animal care committee of Nordrhein-Westfalen (LANUV; Landesamt für Umweltschutz, Naturschutz und Verbraucherschutz Nordrhein-Westfalen, Germany) approved all experiments. Studies were carried out using TRAP mice. Fos-CreER^T2 (+/−)^ × Gt(ROSA)26Sor^tm9(CAG-tdTomato)Hze^/J ^(+/−)^ mice were obtained by crossing Fos-CreER^T2 (+/−)^(RRID: IMSR_JAX:021882) and Gt(ROSA)26Sor^tm9(CAG-tdTomato)Hze^/J ^(+/+)^(RRID: IMSR_JAX:007909) mice. Animals were kept in groups of 2–3 with unlimited access to food and water. Prior to behavioral testing, mice were kept in a separate room with a 12 h light/dark cycle. All tests were conducted during the light phase. Fear conditioning was performed in 5 mice/group of both sexes at 4 to 5 months of age. Mice were habituated to the experimenter for 1 week prior to behavioral testing.

### Drug preparation

4-OHT (Sigma-Aldrich) was dissolved in DMSO and frozen. Shortly before use, 4-OHT was diluted with an 8% Tween 80/saline solution. The final solution was 40 mg/kg 4-OHT and administered intraperitoneally (i.p.) to TRAP mice.

### Fear conditioning

Mice received six pairings consisting of a conditioned stimulus tone (CS) with an unconditioned footshock stimulus (US), in an AB context design during cued fear conditioning as previously described (Batsikadze et al., 2024; Pakusch et al., 2025). Acquisition took place in context A comprising of black and white striped plexiglass walls, white LED illumination and wiped down with 70% ethanol solution. Extinction took place in context B, comprising of gray plexiglass walls, blue LED illumination and wiped down with 1% Helipur solution. The conditioning chamber (23 × 25 × 24 cm) was placed inside a noise-reducing cabinet. A centrally mounted speaker (FR 58 VISATON) delivered the CS. A metallic grid delivered 0.45 mA US to the feet of the animal. The animals were video recorded (Mako U-130B Allied Vision Technologies) to enable post-hoc analysis of fear behavior. A custom MATLAB (The MathWorks) script controlled the timing of tone, shock and video recording.

### TRAP of neurons active during fear acquisition

To investigate active neurons during fear acquisition, the mice were divided into three groups. Mice that underwent fear acquisition (FC) acquired fear memory in context A. The mice had a 2 min baseline period, followed by 6 tone/shock pairings (CS 30 s, 7.5 kHz, 60 dB/US 2 s 0.45 mA co-terminating with the CS). The inter-trial-interval (ITI) ranged from 60 to 180 s. The chamber was thoroughly cleaned between animals. The second group was only subjected to the tone presentation without the shock (NS), while the last group was only subjected to the shock but not the tone (NT). Thirty minutes after the start of the acquisition session, mice were injected i.p. with 4-OHT and returned to their home cage. Twenty-four hours later mice independent of the group were brought to context B for fear extinction (early) starting with a 2 min baseline followed by 10 CS presentations (CS 30 s, 7.5 kHz, 60 dB) alone without the US. The ITIs varied between 30 s and 180 s. Extinction was repeated twice (mid and late extinction).

### Behavior analysis

EthoVision XT 11.5 (Noldus Information Technology) was used to analyze freezing behavior as a readout of fear which was previously described (Batsikadze et al., 2024; Pakusch et al., 2025). To analyze freezing, the changing pixels from one frame to the next were set to a 0.25% threshold to fulfill the criteria of freezing, which is the absence of movement except for respiratory movement for two consecutive seconds. To analyze velocity, the animal’s central position was calculated across frames, and divided by the time duration occurring between these frames. The automated analysis was performed blindly and later verified manually by the researcher. Freezing was analyzed during 30 s CS presentation and baseline activity before CS presentation.

### Histology

Two weeks following the behavior, mice were anesthetized with ketamine/xylazine (100/10 mg/kg) and transcardially perfused with phosphate buffered saline (PBS) followed by 4% paraformaldehyde in PBS (PFA) as previously described (Pakusch et al., 2025). The brains were post-fixed for 4–6 h in PFA and then transferred into 30% sucrose for at least 48 h. Brains were subsequently embedded in Tissue-Tek O. C. T. compound (optimal cutting temperature; Sakura) and 40 μm sagittal cryo-sections (Leica CM3050S) were obtained. Sections were mounted with Mowiol DABCO. Images were acquired using a confocal microscope (Leica Microsystems TCS SP5II). The number of Purkinje cells were determined blindly via live imaging and identified by their morphological properties as well as their localization within the cerebellum (Leica M205 FCA).

### Data visualization and statistical analysis

GraphPad Prism (GraphPad Software, San Diego, California, United States, www.graphpad.com) was used for data visualization and post-processing using CorelDraw^®^ Graphics Suite (Corel Corporation, Ottawa, Canada). Fear behavior is plotted as the mean ± SEM (shaded area). Fear behavior was analyzed using two-way repeated-measures mixed-effects analysis [two-way RM MEA with Geisser–Greenhouse correction (GGC)], as implemented in GraphPad. RM MEA was used to analyze changes in freezing behavior over the course of the trial and between groups, as well as the interaction between groups and trials. Differences in freezing between groups during baseline, retrieval and recall were plotted as boxplots with whiskers representing 10–90 percentiles and were statistically analyzed using two-way RM MEA with GGC, followed by post-hoc Tukey’s multiple comparison test. Neuronal activity was analyzed using two-way RM MEA with GGC with post-hoc multiple comparison (Fisher Least Significant Difference) between groups per lobe.

Velocity is represented as the maximum velocity of the animal during the session or a specific part of the session (baseline, CS or US). Maximum velocity is plotted as boxplots with whiskers representing 10–90 percentiles and were statistically analyzed using two-way RM MEA with GGC with *post hoc* Tukey’s multiple comparison test. Maximum velocity of the groups during specific phases of acquisition were analyzed using two-way RM MEA with GGC followed by post-hoc Tukey’s multiple comparison test.

The PC numbers were counted per lobe in each 40 μm sagittal brain section. Graphical visualization of PC numbers was done as previously mentioned in Pakusch et al., 2025, where PC numbers were counted for each brain and each lobe and subsequently calculated per 10% section by normalizing for the size of each brain (0% being the smallest PC number, 100% the average PC number in all regions of the brain).

## Results

**Figure 1:**
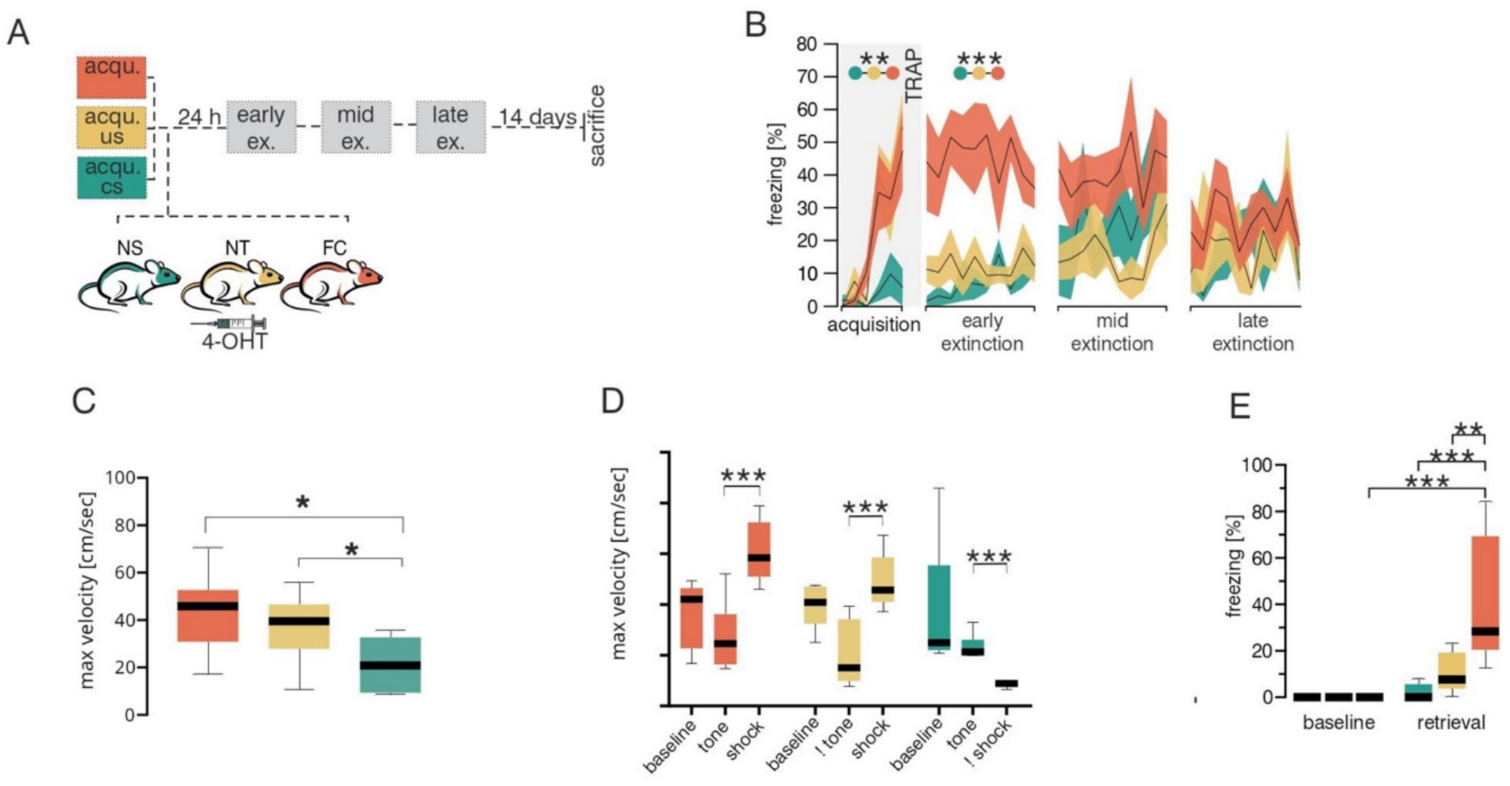
Behavioral data across different groups during fear acquisition. **(A)** Schematic of the experimental design. Mice were divided into three groups for fear acquisition (acqu.): ^1^no shock (unconditioned stimulus, US), tone (conditioned stimulus, CS) only (NS, green), ^2^no tone, shock only (NT, yellow) and classical fear acquisition phase with a paired tone and shock group (FC, orange). Following acquisition all animals were injected with 4-hydroxytamoxifen (4-OHT) and 24 h later subjected to three extinction (ex.; early, mid and late) sessions. **(B)** Percentage freezing behavior during the fear conditioning paradigm for NS (green), NT (yellow) and FC (orange) groups. Shaded areas represent SEMs. **(C)** Boxplots of maximum velocities during acquisition for NS (green), NT (yellow) and FC (orange) groups. **(D)** Boxplots of maximum velocities during specific phases of acquisition such as baseline, tone (CS) and shock (US) for NS (green), NT (yellow) and FC (orange) groups. The Not sign (!) symbolizes only the time where the specific stimulus would arrive but was not given to the animal. In this case, ! tone refers to the time when the tone would have been given, but no tone/CS stimulation was given to the NT group. Similarly in the NS group where the shock/US stimulus was not given but maximum velocities analyzed during this time. **(E)** Boxplots of percentage freezing responses during baseline and retrieval between NS (green), NT (yellow) and FC (orange) groups. Statistical significances indicated by *p ≤ 0.05, **p ≤ 0.01, ***p ≤ 0.001.

**Figure 2:**
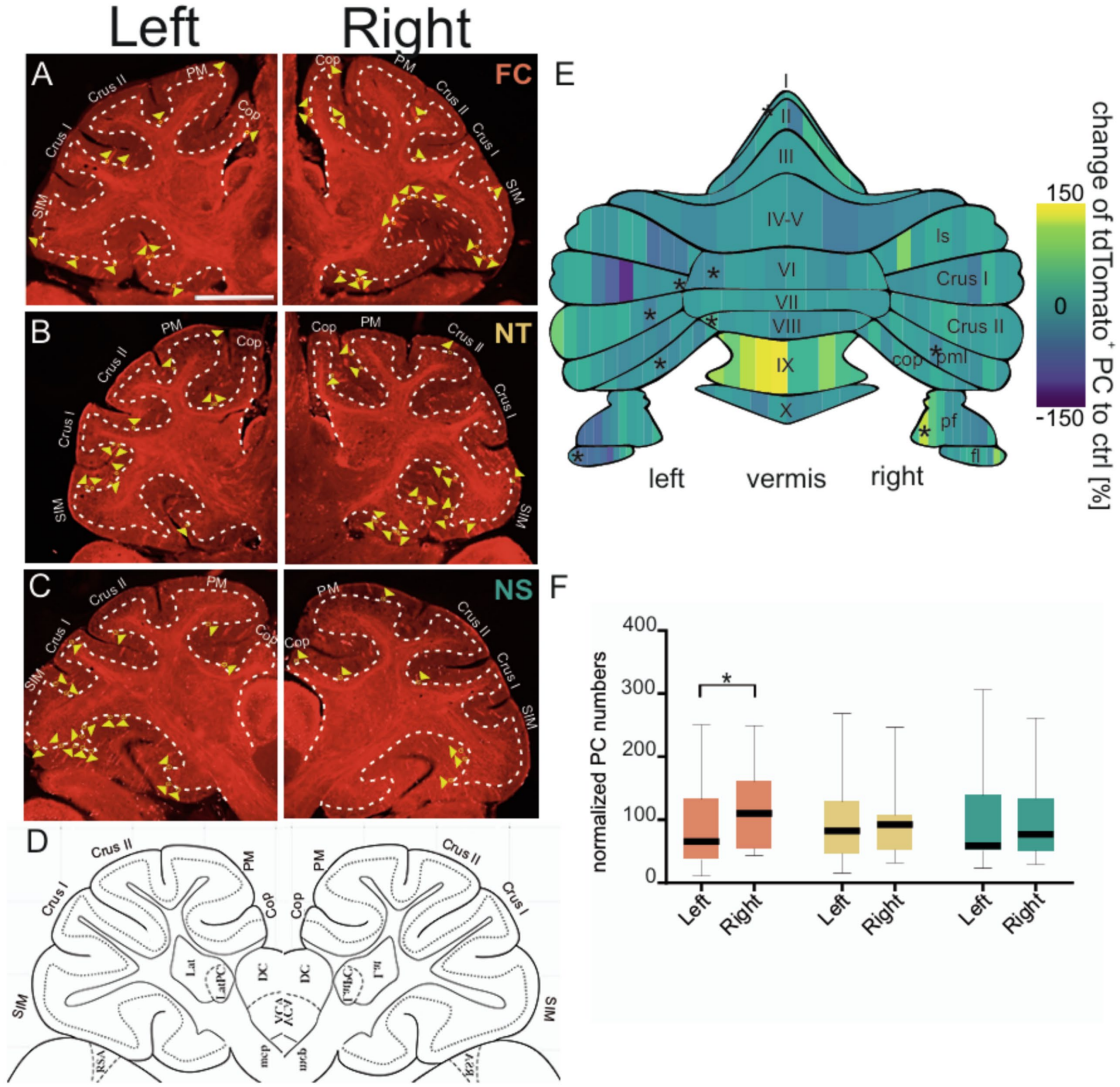
Purkinje cell activity across different cerebellar hemispheres and subregions during fear acquisition and early consolidation. Representative confocal images of sagittal cerebellar slices of the left and right cerebellar hemisphere depicting FosTRAP neurons fluorescing red due to tdTomato in different groups. Example image of cerebellar slice after fear conditioning **(A)** from FC (fear conditioning, orange) group, **(B)** from NT (no tone, yellow) group, and **(C)** from NS (no shock, green) group and **(D)** corresponding sagittal slice from the Allen brain atlas used to identify location and demarcated boundaries (coordinates from bregma: ML: ±2.1, AP: ±6.0, DV: 0.0). Granular cell layer is demarcated using thin continuous white dashes. Active Purkinje cells are depicted by yellow triangles. Sim: simplex lobule; Crus1: Crus 1; Crus2: Crus 2; PM; paramedian lobule; Cop: copula pyramidis. Scale bar is 1 mm. **(E)** Heatmap of normalized Purkinje cell activity (FC corrected to NT and NS controls) divided into 10% bins. The gradient spans from below control group levels in blue to above control levels in yellow. Schematic of the cerebellar flat map was adapted from (Sarpong et al., 2018). ls, lobule simplex; pml, paramedian lobule; pf, paraflocculus; fl, flocculus. **(F)** Boxplots of normalized number of Purkinje cells between the left and right cerebellar hemispheres (vermal region excluded) among FC (orange), NS (green), and NT (yellow) groups. Data represented as mean ± SEM and reported in Supplementary Tables 1–5. Statistical significances indicated by *p ≤ 0.05, **p ≤ 0.01, ***p ≤ 0.001.

*C-fos* is a well-established marker of neuronal activity which has been extensively used to unravel the regions and connections involved in fear learning and extinction. In mice, the TRAP system builds on *c-fos* to selectively and permanently label neurons that are active within a specific time window (Guenthner et al., 2013). TRAP mice were subjected to the classical Pavlovian fear conditioning paradigm where a tone (conditioned stimulus or CS) was paired with a foot shock (unconditioned stimulus or US), and neuronal activity during acquisition was trapped by 4-hydroxytamoxifen (4-OHT; Figure 1A). To control for cerebellar neuronal activity associated with motor coordination or sensory stimuli (e.g., tone, shock), 3 groups of mice were tested: (1) control tone-only group received only the tone but no shock (NS), (2) control shock-only group received only the aversive stimulus but no tone (NT) and (3) fear conditioning (FC) test group underwent a classical fear acquisition by pairing a tone with a shock. Behavioral analysis of fear acquisition (Figure 1B and Supplementary Table 1) revealed significant changes in freezing behavior during acquisition across groups [MEA GGC *F*(2,12) = 8.655, *p* = 0.005]. As expected, the NS group displayed low freezing levels, indicating that the CS alone did not induce fear-like behavior, whereas the FC and NT groups demonstrated elevated freezing responses. In addition to freezing behavior, velocity of the mice in different groups were investigated, to test for any form of movement (such as excessive jumping during the baseline), which might lead to confounding variables in neuronal activation in the control groups. Analyses of maximum velocity (Figure 1C and Supplementary Table 2) revealed an increase in maximum velocity in the FC group [Two way ANOVA *F*(1,8) = 6.640, *p* = 0.0328] and NT group [Two way ANOVA *F*(1,8) = 11.16, *p* = 0.0102] in compairson to the NS group. To further investigate whether the increase in maximum velocity in FC and NS groups are occuring during the US initiation period, we compared the maximum velocity among the three groups during distinct phases of the paradigm, including baseline, CS and US (Figure 1D and Supplementary Table 3). As expected the maximum velocity was higher during the US phase of the trials in the FC and NT groups, whereas they were lower in the NS group, indicating that high velocity movements were linked to shock onset and not to other phases of the trial. It is important to note that the maximum velocity during tone depicted in the NT group, is only to indicate the velocity during similar duration and timepoint the tone would have been given in the corresponding FC group, however, in the case of the NT no actual CS was presented to the animal. Similar trend applies to the US/shock in the NS groups. Twenty-four hours later, fear retrieval was assessed by exposing the TRAP mice to the tone alone in a novel context. Only the FC group displayed elevated freezing compared to baseline levels (*post hoc* Tukey *p* ≤ 0.001), whereas both control groups showed no fear behavior during fear retrieval (Figure 1E and Supplementary Table 4). Extinction learning was conducted for three consecutive days, confirming that only the FC group formed an associative fear memory specific to the tone (Figure 1B and Supplementary Table 1). After acquisition TRAP mice from different groups were injected with 4-OHT to evaluate the PC activity (Figures 2A–D) formed during fear acquisition which was normalized to the combined activity of both control groups and split into 10% bins for each lobule (Figure 2E and Table 1). The effect of lobules was significant, *F*(7.784, 93.69) = 14.49, *p* = <0.001, partial η^2^ = 0.55 (Cohen’s *f* = 1.10, ω^2^ = 0.53), indicating a large effect. Further comparison using Fisher’s Least Significant Difference test depicted PC activity was predominantly decreased in the left cerebellar hemispheric subregions of specific lobules I and VI, as well as the specific lobules left flocculus, copula pyramidis, Crus I and Crus II and in the right paramedian lobule. We also detected increased PC activity in sub lobules of the right paraflocculus and the left lobule VIII. To test for hemispheric lateralization, the normalized number of PCs was compared between the left and right hemispheres across all groups. There was no hemispheric effect across the groups, *F*(2,95) = 1.726, *p* = 0.183. However, Tukey’s multiple comparison depicted a decrease in the left hemispheric PC activity from the FC groups, whereas PC activity from the controls (NS and NT), did not differ between the two hemispheres (Figure 2F and Supplementary Table 5).

**Table 1:** TRAP during fear acquisition detailed PC activity.

Acquisition Fisher’s LSD normalized PC (heatmap)				
Lobule	% of lobule from right to left	Mean diff.	95.00% CI of diff.	*p*-value
pf right	10%	24.56	−103.6 to 152.7	0.609
	20%	−39.23	−120.2 to 41.74	0.287
	30%	−33.31	−120.0 to 53.35	0.410
	40%	−24.76	−84.29 to 34.77	0.365
	50%	−10.54	−102.7 to 81.62	0.756
	60%	−12.92	−99.33 to 73.50	0.713
	70%	32.40	−124.9 to 189.7	0.614
	80%	61.02	−44.11 to 166.1	0.211
	90%	120.9	18.00 to 223.7	**0.030**
	100%	37.91	−47.50 to 123.3	0.349
fl right	10%	48.13	−239.1 to 335.4	0.638
	20%	95.19	−263.9 to 454.2	0.468
	30%	−15.95	−246.0 to 214.1	0.853
	40%	19.73	−172.4 to 211.9	0.785
	50%	1.049	−289.1 to 291.2	0.993
	60%	29.50	−223.2 to 282.2	0.774
	70%	22.42	−202.9 to 247.7	0.799
	80%	−5.272	−175.0 to 164.4	0.942
	90%	6.935	−154.6 to 168.5	0.915
	100%	−19.31	−133.8 to 95.23	0.695
cop right	10%	−4.302	−42.15 to 33.55	0.781
	20%	−11.52	−41.30 to 18.26	0.385
	30%	7.412	−21.12 to 35.94	0.573
	40%	23.95	−15.30 to 63.20	0.181
	50%	2.462	−28.43 to 33.35	0.852
	60%	12.06	−31.26 to 55.39	0.515
	70%	15.46	−28.24 to 59.17	0.420
	80%	−21.07	−48.65 to 6.504	0.118
	90%	32.90	−72.39 to 138.2	0.402
	100%	−13.68	−80.79 to 53.43	0.616
pml right	10%	−5.364	−36.66 to 25.94	0.677
	20%	28.84	−56.62 to 114.3	0.396
	30%	−16.73	−50.20 to 16.74	0.294
	40%	−10.36	−41.08 to 20.37	0.477
	50%	4.200	−37.48 to 45.88	0.816
	60%	−31.51	−54.77 to −8.241	**0.012**
	70%	0.3144	−31.50 to 32.13	0.983
	80%	6.159	−24.68 to 36.99	0.663
	90%	18.18	−19.47 to 55.83	0.248
	100%	−8.995	−37.93 to 19.94	0.474
Crus II right	10%	−0.6395	−46.56 to 45.28	0.973
	20%	1.353	−60.55 to 63.26	0.956
	30%	5.593	−81.03 to 92.22	0.868
	40%	1.850	−71.43 to 75.13	0.950
	50%	−4.215	−45.99 to 37.56	0.817
	60%	30.74	−45.68 to 107.1	0.297
	70%	20.68	−30.27 to 71.63	0.326
	80%	−1.283	−51.95 to 49.39	0.947
	90%	−10.56	−43.51 to 22.39	0.417
	100%	8.749	−15.07 to 32.57	0.379
Crus I right	10%	−0.3480	−36.39 to 35.69	0.983
	20%	−1.479	−68.63 to 65.67	0.962
	30%	28.48	−63.10 to 120.1	0.507
	40%	−45.16	−175.2 to 84.90	0.462
	50%	−16.58	−154.4 to 121.2	0.789
	60%	−13.18	−170.4 to 144.0	0.836
	70%	−3.165	−75.24 to 68.91	0.921
	80%	−13.53	−57.84 to 30.78	0.511
	90%	−17.82	−49.88 to 14.24	0.246
	100%	−15.87	−47.50 to 15.76	0.294
ls right	10%	6.439	−107.7 to 120.5	0.881
	20%	21.61	−53.09 to 96.31	0.480
	30%	18.60	−61.96 to 99.16	0.594
	40%	−3.973	−108.3 to 100.4	0.934
	50%	12.91	−128.6 to 154.4	0.828
	60%	19.09	−55.27 to 93.46	0.581
	70%	−2.612	−98.09 to 92.86	0.947
	80%	84.21	−62.25 to 230.7	0.196
	90%	−1.721	−80.16 to 76.72	0.958
	100%	5.916	−60.11 to 71.94	0.831
X	10%	−6.345	−26.56 to 13.87	0.506
	20%	−11.05	−44.90 to 22.81	0.449
	30%	−3.366	−76.77 to 70.03	0.919
	40%	−14.97	−109.6 to 79.61	0.712
	50%	−23.74	−192.9 to 145.4	0.721
	60%	3.400	−122.7 to 129.5	0.941
	70%	−38.62	−139.7 to 62.49	0.366
	80%	−6.039	−63.40 to 51.32	0.809
	90%	5.286	−94.13 to 104.7	0.879
	100%	−18.17	−65.76 to 29.43	0.369
IX	10%	42.44	−107.1 to 192.0	0.448
	20%	47.24	−205.0 to 299.5	0.601
	30%	90.03	−199.7 to 379.8	0.403
	40%	34.01	−71.00 to 139.0	0.409
	50%	32.99	−98.41 to 164.4	0.525
	60%	147.9	−289.9 to 585.8	0.365
	70%	140.6	−254.5 to 535.6	0.346
	80%	114.3	−221.0 to 449.6	0.362
	90%	71.91	−96.02 to 239.8	0.277
	100%	102.4	−259.4 to 464.2	0.437
VIII	10%	5.020	−26.67 to 36.71	0.716
	20%	−15.16	−63.30 to 32.97	0.505
	30%	−8.087	−96.68 to 80.51	0.843
	40%	−16.37	−122.5 to 89.79	0.739
	50%	−19.71	−96.79 to 57.37	0.567
	60%	−24.19	−88.65 to 40.28	0.425
	70%	−1.145	−101.1 to 98.77	0.978
	80%	0.5139	−26.85 to 27.88	0.968
	90%	−7.699	−34.15 to 18.75	0.516
	100%	36.27	13.60 to 58.95	0.**005**
VII	10%	−8.536	−27.52 to 10.45	0.346
	20%	−5.664	−15.68 to 4.357	0.223
	30%	−0.3787	−14.73 to 13.97	0.946
	40%	−1.829	−6.702 to 3.044	0.387
	50%	8.752	−4.827 to 22.33	0.146
	60%	3.040	−12.43 to 18.51	0.622
	70%	−3.018	−7.187 to 1.150	0.140
	80%	6.403	−5.073 to 17.88	0.199
	90%	11.73	−3.676 to 27.13	0.109
	100%	−2.724	−22.47 to 17.02	0.768
VI	10%	13.36	−36.94 to 63.66	0.547
	20%	−1.296	−41.32 to 38.73	0.942
	30%	−25.89	−85.13 to 33.35	0.347
	40%	−14.44	−45.77 to 16.88	0.334
	50%	3.244	−56.97 to 63.46	0.891
	60%	−14.57	−40.11 to 10.97	0.221
	70%	−2.592	−50.17 to 44.99	0.890
	80%	0.2138	−22.58 to 23.00	0.984
	90%	−24.68	−46.88 to −2.480	**0.032**
	100%	−24.35	−67.35 to 18.65	0.240
IV/V	10%	−20.05	−58.64 to 18.54	0.244
	20%	−21.80	−59.45 to 15.85	0.217
	30%	−30.16	−79.05 to 18.73	0.193
	40%	−44.12	−115.2 to 26.91	0.193
	50%	−10.42	−122.2 to 101.4	0.809
	60%	0.8506	−99.09 to 100.8	0.984
	70%	−5.993	−142.5 to 130.5	0.913
	80%	−26.00	−95.81 to 43.80	0.424
	90%	−13.52	−67.98 to 40.93	0.598
	100%	−21.88	−80.05 to 36.29	0.426
III	10%	−11.54	−40.16 to 17.09	0.391
	20%	−11.25	−33.64 to 11.15	0.289
	30%	−2.033	−30.27 to 26.20	0.872
	40%	6.339	−52.47 to 65.15	0.802
	50%	0.7834	−59.49 to 61.05	0.977
	60%	−3.929	−122.2 to 114.3	0.937
	70%	−2.707	−95.34 to 89.92	0.943
	80%	−21.10	−77.52 to 35.32	0.403
	90%	−8.705	−42.81 to 25.40	0.588
	100%	1.348	−48.30 to 50.99	0.949
II	10%	15.01	−20.05 to 50.06	0.318
	20%	41.05	−23.21 to 105.3	0.166
	30%	38.82	−68.94 to 146.6	0.392
	40%	41.55	−51.71 to 134.8	0.298
	50%	−43.55	−119.1 to 32.01	0.205
	60%	9.557	−50.32 to 69.43	0.722
	70%	16.65	−28.55 to 61.85	0.425
	80%	−6.088	−70.30 to 58.12	0.834
	90%	−21.08	−55.75 to 13.58	0.187
	100%	−1.219	−34.04 to 31.60	0.927
I	10%	−0.7371	−16.41 to 14.94	0.913
	20%	−11.56	−35.55 to 12.42	0.275
	30%	19.18	−34.59 to 72.94	0.364
	40%	37.45	−20.50 to 95.39	0.148
	50%	22.64	−60.40 to 105.7	0.465
	60%	0.2089	−42.78 to 43.20	0.990
	70%	−13.15	−42.66 to 16.37	0.305
	80%	−13.35	−26.14 to −0.5529	**0.043**
	90%	−6.609	−27.94 to 14.73	0.456
	100%	−5.804	−15.34 to 3.731	0.209
pf left	10%	33.08	−67.50 to 133.7	0.485
	20%	−5.990	−95.09 to 83.10	0.886
	30%	7.539	−102.0 to 117.1	0.881
	40%	5.153	−76.62 to 86.92	0.890
	50%	26.55	−53.75 to 106.8	0.485
	60%	−15.27	−91.88 to 61.34	0.672
	70%	−30.39	−116.8 to 56.00	0.458
	80%	−69.95	−192.4 to 52.49	0.231
	90%	−58.08	−148.5 to 32.29	0.182
	100%	−62.85	−165.5 to 39.85	0.204
fl left	10%	44.24	−155.1 to 243.6	0.546
	20%	18.98	−219.9 to 257.9	0.830
	30%	−22.08	−206.4 to 162.2	0.751
	40%	−90.58	−205.2 to 24.05	0.106
	50%	−62.70	−185.8 to 60.40	0.276
	60%	−52.19	−174.7 to 70.27	0.350
	70%	−60.91	−164.5 to 42.63	0.214
	80%	−59.83	−198.2 to 78.57	0.313
	90%	−64.63	−94.77 to −34.49	**<0.001**
	100%	−21.52	−45.56 to 2.526	0.074
cop left	10%	14.19	−39.77 to 68.16	0.525
	20%	−27.67	−81.24 to 25.91	0.265
	30%	−5.350	−54.31 to 43.60	0.789
	40%	−23.08	−62.65 to 16.50	0.199
	50%	−20.78	−39.55 to −2.009	**0.033**
	60%	−3.573	−48.68 to 41.54	0.832
	70%	−10.66	−53.70 to 32.38	0.535
	80%	−16.54	−48.01 to 14.94	0.258
	90%	13.63	−49.84 to 77.10	0.563
	100%	16.05	−21.22 to 53.32	0.313
pm left	10%	−5.401	−51.93 to 41.13	0.767
	20%	−3.621	−77.68 to 70.44	0.894
	30%	−28.71	−76.31 to 18.89	0.170
	40%	−17.61	−74.64 to 39.43	0.422
	50%	−16.19	−63.86 to 31.48	0.413
	60%	−24.41	−75.13 to 26.30	0.247
	70%	−14.29	−49.27 to 20.69	0.335
	80%	0.3829	−44.95 to 45.72	0.984
	90%	23.02	−1.999 to 48.03	0.068
	100%	17.08	−27.46 to 61.63	0.351
Crus II left	10%	−7.840	−28.51 to 12.83	0.403
	20%	−8.138	−32.00 to 15.72	0.440
	30%	−36.57	−71.34 to −1.810	**0.042**
	40%	−5.847	−67.10 to 55.41	0.815
	50%	23.87	−25.99 to 73.72	0.266
	60%	14.73	−45.29 to 74.75	0.541
	70%	22.19	−60.66 to 105.0	0.497
	80%	4.840	−29.85 to 39.53	0.766
	90%	31.50	−57.77 to 120.8	0.397
	100%	73.63	−38.26 to 185.5	0.138
Crus I left	10%	−19.73	−39.41 to −0.04160	**0.050**
	20%	−7.182	−49.17 to 34.80	0.665
	30%	−15.12	−62.63 to 32.40	0.467
	40%	−7.571	−95.75 to 80.61	0.842
	50%	−123.1	−299.0 to 52.70	0.131
	60%	−75.90	−368.1 to 216.3	0.514
	70%	−47.43	−369.0 to 274.1	0.706
	80%	20.65	−347.6 to 388.9	0.879
	90%	18.47	−109.8 to 146.7	0.730
	100%	5.017	−98.75 to 108.8	0.915
ls left	10%	5.172	−42.85 to 53.19	0.817
	20%	−43.10	−145.4 to 59.20	0.368
	30%	−46.55	−234.7 to 141.6	0.528
	40%	−60.42	−197.0 to 76.12	0.276
	50%	10.58	−123.6 to 144.7	0.853
	60%	16.12	−142.5 to 174.7	0.796
	70%	−12.29	−53.52 to 28.94	0.528
	80%	−10.95	−67.30 to 45.40	0.678
	90%	20.38	−24.75 to 65.51	0.323
	100%	8.655	−24.51 to 41.82	0.573

Bold values indicate statistical significance.

## Discussion

The complex structure of the brain reveals, how different subregions within these areas, interact and play distinct roles. In line with this, we found that specific subregions within the cerebellar cortex displayed altered PC activity during fear conditioning in mice. A comprehensive interpretation of our findings highlights the greater contribution of the left cerebellar hemisphere including subregions of Crus I, Crus II and lobule VI during processing of fear emotions.

During fear acquisition we observed changes in PC activity in multiple subregions of the seven lobules. While the majority were predominantly in the left, two of these regions were in the right cerebellar hemisphere. Most likely the observed changes in activity in lobule I, flocculus, paraflocculus and copula pyramidis are due to their involvement in motor coordination and balance in response to the tone, and especially to the electrical shock, as indicated by higher velocity of the animal during the shock phases of the trial. Copula pyramidis has been shown to be responsive to hindlimb shocks, which are likely driven by climbing fiber inputs (Lawrenson et al., 2016). The other three regions have been linked to their role in cognition. For example, Crus 1 and lobule VI has been previously implicated in fear conditioning, especially when the CS is linked to the US, suggesting their role in predicting aversive events (Ernst et al., 2019). Furthermore, PC activity changes in lobule VI are critical for the process of fear consolidation (Sacchetti et al., 2004). Finally, in our fear conditioning paradigm, the inter-trial intervals varied, while the shock consistently arrived 28 s after the start of the tone. Since Crus II is known to be involved in absolute timing processes, this might explain the altered activity pattern in Crus II during fear acquisition in PCs (Yamaguchi and Sakurai, 2016). TRAPing the active neurons is a dynamic process which can extend beyond the specific time frame of the behavioral paradigm. *C-fos* activity in mice has been demonstrated to peak between 60 to 120 min after stimulation whereas the half-life of 4-OHT in mice is around 6 h (Lara Aparicio et al., 2022; Brandhorst et al., 2024). Based on these estimates, our results most likely not only capture neurons active during fear acquisition but also the initial phases of memory consolidation. In line with this activity changes in the cognitive regions of lobule VI could likely be associated with early memory consolidation processes in addition to fear acquisition. Our findings suggest that cerebellar lobule subregions may be involved in processing sensorimotor information, as well as consolidation information and their integration into the existing fear learning network.

Combining the obtained results imply that these effects are specifically prevalent in disrupting learning through lobules VIII or Crus I, as they receive and send information to fear-related brain areas and may extend learning impairments from the cerebellum to the whole network. Additionally, information is processed with a left-sided dominance in the cerebellar cortex before being integrated into the right-dominant areas of the cerebral fear network.

Evidence regarding cerebellar lateralization during fear learning can be derived from human fMRI studies which directly assess hemispheric differences and a recent rodent study. In humans the cerebral fear network is driven by the right hemisphere which agrees with the contralateral activation of the cerebellar left hemisphere. During acquisition, unexpected US removal resulted in cerebellar activation with a localized hotspot of activity in Crus I and VI, extending to Crus II. Extinction learning makes the omission more predictable and expected, which reduces activation in the left Crus I (Ernst et al., 2019). In the initial extinction phase also known as fear retrieval, where the CS is associated to predict the US, lobule VI displays heightened activity upon the unexpected US omission. Lobule VI and Crus I activation were also detected during early extinction in humans (Ernst et al., 2019; Batsikadze et al., 2022; Nio et al., 2025). In agreement with the human studies supporting cerebellar lateralization of fear emotions, a recent TRAP study in mice demonstrated augmented granule cell layer, the main driver of fMRI activity, around lobule simplex, Crus I/II and lobule VI predominantly in the left hemisphere during early extinction (Pakusch et al., 2025). Lateralized extinction related Purkinje cell activity was demonstrated in the left cerebellar subregions of lobule simplex and Crus II in TRAP mice since PC activity cannot be measured in fMRI studies, indicating that the left hemisphere may be contributing to the cerebellar lateralization of fear emotions during early extinction (Pakusch et al., 2025). Initially the overall activity levels appeared to be bilaterally distributed, however, flatmap representations display more pronounced activity in the left hemisphere. Similar cerebellar lateralization effects were also observed in this study, except a decrease in the number of active Purkinje cells were evident in the left hemisphere of Crus I/II and lobule VI.

The findings of this study, however, do have potential limitations. The effects described in the study, are limited by their small sample size. Due to considerable variability present within the groups, the effects observed maybe mild and occurring in a smaller subpopulation of the cerebellar lobule, which may underestimate the accurate underlying effects, leaning toward a limited generalizability of these findings. Further research focusing on larger samples may help explore these effects more in depth. Furthermore, to reduce untoward within group variability, an unpaired fear conditioned group, employing a gap between the tone and shock, can help curtail sensory driven variability, which was observed when using two different groups for shock and tone separately. Moreover, future studies including TRAP changes from all fear network brain regions such as the amygdala, periaqueductal gray area, medial prefrontal cortex, ventral tegmental area, hippocampus and dorsal striatum in a purely cerebellar degenerative compared to control mouse lines would strengthen our cerebellar lateralization of fear emotions studies.

Despite these certain limitations, we are the first to report a decrease in *c-fos*-driven PC activity during acquisition and early consolidation. Our findings are in line with studies depicting the involvement of similar cerebellar structures during fear conditioning in mice as well as fMRI studies in humans. Moreover, our findings lay a foundation in understanding and further investigating the role of cerebellar lateralization, specifically in cognition and fear learning, while also highlighting the necessity of examining the involvement of an area beyond the broad segmentation and recognizing and valuing the contribution of subregions at a hemispheric level.

## Data Availability

Data needed to evaluate the conclusions in this manuscript are present in the manuscript or supplementary material. Additional inquiries will be provided by the corresponding author at melanie.mark@rub.de.
