# Supplementary material for: Purkinje cell activity changes in cerebellar subregions during fear conditioning
Source: Front Behav Neurosci. 2025 Nov 12;19:1649361. doi: 10.3389/fnbeh.2025.1649361 (PMC12647051; doi:10.3389/fnbeh.2025.1649361)
Supplement: Supplementary file 1 [file Data_Sheet_1.docx]

***Supplementary Table 1:TRAP during fear acquisition behavior***

| ***FC/NT/NS*** | **Factor** | **Num Df** | **Den Df** | ***F*** | ***P*** | |
| --- | --- | --- | --- | --- | --- | --- |
| **TRAP fear acquisition MEA** | | | | | | |
| *fear acquisition training* | | | | | | |
| 5/5/5 | Trial  Group  Trial × group | 3.034  2  10 | 36.41  12  60 | 16.23  8.655  3.080 | | **< .001**  **0.005**  **0.003** |
| *early extinction training* | | | | | | |
| 5/5/5 | Trial  Group  Trial × group | 2.373  2  18 | 28.47  12  108 | 0.3986  14.08  1.049 | | 0.709  **0.001**  0.413 |
| *mid extinction training* | | | | | | |
| 5/5/5 | Trial  Group  Trial × group | 2.283  2  18 | 27.39  12  108 | 1.716  2.414  1.297 | | 0.195  0.131  0.204 |
| *late extinction training* | | | | | | |
| 5/5/5 | Trial  Group  Trial × group | 3.905  2  18 | 46.86  12  108 | 1.662  1.129  0.5955 | | 0.176  0.355  0.896 |

***Supplementary Table 2: Maximum velocity during fear acquisition behavior***

| ***FC/ NT/NS*** | **Factor** | **Num Df** | **Den Df** | ***F*** | ***P*** | |
| --- | --- | --- | --- | --- | --- | --- |
| ***TRAP fear acquisition maximum velocity two-way RM ANOVA*** | | | | | |  |
| 5/5/5 | Trial  Group  Trial × Groups | 3.410  2  6.8321 | 40.93  12  40.93 | 2.440  4.417  2.424 | 0.0709  **0.0365**  **0.0368** | |
| **Post-hoc test (Turkey’s multiple comparison)**   \| **Group** \| \| **Trials** \| **Significance *p*** \| \| \| \| \| --- \| --- \| --- \| --- \| --- \| --- \| --- \| \| FC x NT \| Baseline \| \| \| ns \| *0,8526* \| \| FC x NS \| Baseline \| \| \| ns \| *>0,9999* \| \| NT x NS \| Baseline \| \| \| ns \| *0,9503* \| \| FC x NT \| CS1 \| \| \| ns \| *0,8174* \| \| FC x NS \| CS1 \| \| \| ns \| *0,4207* \| \| NT x NS \| CS1 \| \| \| ns \| *0,2879* \| \| FC x NT \| US1 \| \| \| ns \| *0,7987* \| \| FC x NS \| US1 \| \| \| ****** \| ***0,0052*** \| \| NT x NS \| US1 \| \| \| ******** \| ***<0,0001*** \| \| FC x NT \| ITI1 \| \| \| ns \| *0,5847* \| \| FC x NS \| ITI1 \| \| \| ns \| *0,9731* \| \| NT x NS \| ITI1 \| \| \| ns \| *0,9261* \| \| FC x NT \| CS2 \| \| \| ns \| *0,7365* \| \| FC x NS \| CS2 \| \| \| ns \| *0,6454* \| \| NT x NS \| CS2 \| \| \| ns \| *0,8275* \| \| FC x NT \| US2 \| \| \| ns \| *0,9952* \| \| FC x NS \| US2 \| \| \| ns \| *0,0696* \| \| NT x NS \| US2 \| \| \| ******** \| ***<0,0001*** \| \| FC x NT \| ITI2 \| \| \| ns \| *0,4976* \| \| FC x NS \| ITI2 \| \| \| ns \| *0,5196* \| \| NT x NS \| ITI2 \| \| \| ns \| *0,9982* \| \| FC x NT \| CS3 \| \| \| ns \| *0,9233* \| \| FC x NS \| CS3 \| \| \| ns \| *0,4625* \| \| NT x NS \| CS3 \| \| \| ns \| *0,5221* \| \| FC x NT \| US3 \| \| \| ns \| *0,1464* \| \| FC x NS \| US3 \| \| \| ***** \| ***0,0127*** \| \| NT x NS \| US3 \| \| \| ****** \| ***0,0045*** \| \| FC x NT \| ITI3 \| \| \| ns \| *0,8827* \| \| FC x NS \| ITI3 \| \| \| ns \| *0,3549* \| \| NT x NS \| ITI3 \| \| \| ns \| *0,2685* \| \| FC x NT \| CS4 \| \| \| ***** \| ***0,0315*** \| \| FC x NS \| CS4 \| \| \| ns \| *0,7230* \| \| NT x NS \| CS4 \| \| \| ***** \| ***0,0215*** \| \| FC x NT \| US4 \| \| \| ns \| *0,9998* \| \| FC x NS \| US4 \| \| \| ns \| *0,0947* \| \| NT x NS \| US4 \| \| \| ***** \| ***0,0164*** \| \| FC x NT \| ITI4 \| \| \| ns \| *0,9624* \| \| FC x NS \| ITI4 \| \| \| ns \| *0,8381* \| \| NT x NS \| ITI4 \| \| \| ns \| *0,4018* \| \| FC x NT \| CS5 \| \| \| ns \| *0,3550* \| \| FC x NS \| CS5 \| \| \| ns \| *0,5806* \| \| NT x NS \| CS5 \| \| \| ns \| *0,0852* \| \| FC x NT \| US5 \| \| \| ns \| *0,5516* \| \| FC x NS \| US5 \| \| \| ns \| *0,1908* \| \| NT x NS \| US5 \| \| \| ns \| *0,0845* \| \| FC x NT \| ITI5 \| \| \| ns \| *0,7599* \| \| FC x NS \| ITI5 \| \| \| ns \| *0,8776* \| \| NT x NS \| ITI5 \| \| \| ns \| *0,6036* \| \| FC x NT \| CS6 \| \| \| ns \| *0,5870* \| \| FC x NS \| CS6 \| \| \| ns \| *0,8227* \| \| NT x NS \| CS6 \| \| \| ns \| *0,6663* \| \| FC x NT \| US6 \| \| \| ns \| *0,9019* \| \| FC x NS \| US6 \| \| \| ns \| *0,1268* \| \| NT x NS \| US6 \| \| \| ns \| *0,2272* \| \| FC x NT \| Post trial interval \| \| \| ns \| *0,9908* \| \| FC x NS \| Post trial interval \| \| \| ns \| *0,4809* \| \| NT x NS \| Post trial interval \| \| \| ns \| *0,5437* \|   ***Supplementary Table 3: Maximum velocity during fear acquisition behavior during specific time periods of the paradigm***   \| ***FC/ NT/NS*** \| **Factor** \| **Num Df** \| **Den Df** \| ***F*** \| ***P*** \| \| \| --- \| --- \| --- \| --- \| --- \| --- \| --- \| \| ***TRAP fear acquisition maximum velocity two way RM MEM ANOVA*** \| \| \| \| \| \| \| 5/5/5 \| Trial (Baseline x CS x US)  Group  Trial × Groups \| 2  1.636  3.271 \| 186  152.1  152.1 \| 10.98  5.868  10.53 \| **<0.0001**  **0.0061**  **<0.0001** \| \| \| **Post-hoc test (Turkey’s multiple comparison)**   \| **Group** \| \| **Trials** \| **Significance *p*** \| \| \| \| \| --- \| --- \| --- \| --- \| --- \| --- \| --- \| \| FC x NT \| Baseline \| \| \| ns \| *0,8526* \| \| FC x NS \| Baseline \| \| \| ns \| *>0,9999* \| \| NT x NS \| Baseline \| \| \| ns \| *0,9503* \| \| FC x NT \| CS \| \| \| ns \| *0,8174* \| \| FC x NS \| CS \| \| \| ns \| *0,4207* \| \| NT x NS \| CS \| \| \| ns \| *0,2879* \| \| FC x NT \| US \| \| \| ns \| *0,7987* \| \| FC x NS \| US \| \| \| ******** \| ***<0,0001*** \| \| NT x NS \| US \| \| \| ******** \| ***<0,0001*** \| \| Baseline x CS \| FC \| \| \| ns \| *0,5847* \| \| Baseline x US \| FC \| \| \| ***** \| ***0,0342*** \| \| CS x US \| FC \| \| \| ******* \| ***0,0007*** \| \| Baseline x CS \| NT \| \| \| ****** \| ***0,0093*** \| \| Baseline x US \| NT \| \| \| ns \| *0,6454* \| \| CS x US \| NT \| \| \| ******** \| ***<0,0001*** \| \| Baseline x CS \| NS \| \| \| ns \| *0,9952* \| \| Baseline x US \| NS \| \| \| ns \| *0,0696* \| \| CS x US \| NS \| \| \| ******** \| ***<0,0001*** \| \|  \|  \| \| \|  \|  \| \| \| \| \| \| \| \| | | | | | | |

***Supplementary Table 4: TRAP during fear acquisition behavior retrieval***

| ***FC/ NT/NS*** | **Factor** | **Num Df** | **Den Df** | ***F*** | ***P*** | |
| --- | --- | --- | --- | --- | --- | --- |
| ***TRAP fear acquisition baseline x retrieval MEA*** | | | | | |  |
| 5/5/5 | Trial (Baseline x Retrieval)  Group  Trial × Genotype | 1  2  2 | 24  24  24 | 6.88  7.262  7.262 | **<.001**  0.003  0.003 | |
| **Post-hoc test (Turkey)**   \| **Group** \| **Trial** \| ***p*** \| \| --- \| --- \| --- \| \| FC \| Baseline x Retrieval \| **<.001** \| \| NS \| Baseline x Retrieval \| 0.767 \| \| NT \| Baseline x Retrieval \| 0.175 \| \| FC x NS \| Baseline \| >.999 \| \| FC x NT \| Baseline \| >.999 \| \| NS x NT \| Baseline \| >.999 \| \| FCx NS \| Retrieval \| **<.001** \| \| FC x NT \| Retrieval \| **.001** \| \| NS x NT \| Retrieval \| .524 \| | | | | | |  |

| **Factor** | **Num Df** | **Den Df** | ***F*** | ***p*** |  |
| --- | --- | --- | --- | --- | --- |
| ***TRAP fear acquisition baseline x retrieval MEA*** | | | | | |
| Hemisphere (Left/Right)  Group (FC/NT/NS)  Hemisphere x Group | 1  2  2 | 95  95  95 | 2.561  0.026  1.726 | 0.112  0.973  0.183 |  |
| **Post-hoc test (Turkey’s multiple comparison test)**   \| **Group** \| **Trial** \| ***p*** \| \| --- \| --- \| --- \| \| FC \| Right hemisphere x Left hemisphere \| ***0.023*** \| \| NS \| Right hemisphere x Left hemisphere \| 0.723 \| \| NT \| Right hemisphere x Left hemisphere \| 0.961 \| \| Right hemisphere \| FC x NS \| 0.894 \| \| Right hemisphere \| FC x NT \| 0.761 \| \| Right hemisphere \| NS x NT \| 0.960 \| \| Left hemisphere \| FC x NS \| 0.909 \| \| Left hemisphere \| FC x NT \| 0.945 \| \| Left hemisphere \| NS x NT \| 0.994 \| | | | | | |

***Supplementary Table 5: Number of Purkinje cells between hemispheres across groups***
